# Human Complement Regulators C4b-Binding Protein and C1 Esterase Inhibitor Interact with a Novel Outer Surface Protein of *Borrelia recurrentis*

**DOI:** 10.1371/journal.pntd.0000698

**Published:** 2010-06-01

**Authors:** Sonja Grosskinsky, Melanie Schott, Christiane Brenner, Sally J. Cutler, Markus M. Simon, Reinhard Wallich

**Affiliations:** 1 Infectious Immunology Group, Institute for Immunology, University of Heidelberg, Heidelberg, Germany; 2 School of Health and Bioscience, University of East London, Stratford, London, United Kingdom; University of Washington, United States of America

## Abstract

The spirochete *Borrelia recurrentis* is the causal agent of louse-borne relapsing fever and is transmitted to humans by the infected body louse *Pediculus humanus*. We have recently demonstrated that the *B. recurrentis* surface receptor, HcpA, specifically binds factor H, the regulator of the alternative pathway of complement activation, thereby inhibiting complement mediated bacteriolysis. Here, we show that *B. recurrentis* spirochetes express another potential outer membrane lipoprotein, termed CihC, and acquire C4b-binding protein (C4bp) and human C1 esterase inhibitor (C1-Inh), the major inhibitors of the classical and lectin pathway of complement activation. A highly homologous receptor for C4bp was also found in the African tick-borne relapsing fever spirochete *B. duttonii*. Upon its binding to *B. recurrentis* or recombinant CihC, C4bp retains its functional potential, i.e. facilitating the factor I-mediated degradation of C4b. The additional finding that ectopic expression of CihC in serum sensitive *B. burgdorferi* significantly increased spirochetal resistance against human complement suggests this receptor to substantially contribute, together with other known strategies, to immune evasion of *B. recurrentis*.

## Author Summary

*Borrelia recurrentis,* the causal agent of louse-borne relapsing fever is transmitted to humans via infected body lice. Infection with *B. recurrentis* has been achieved only in humans and is accompanied by a systemic inflammatory disease, multiple relapses of fever and massive spirochetemia. A key virulence factor of *B. recurrentis* is their potential to undergo antigenic variation. However, for survival in the blood during the early phase of infection and for persistence in human tissues, spirochetes must be endowed with robust tools to escape innate immunity. We have recently shown that *B. recurrentis* acquires the serum-derived regulator factor H, thereby blocking the alternative complement pathway. Here, we show that *B. recurrentis* expresses in addition a novel outer surface lipoprotein that selectively binds serum-derived C4b-binding protein and C1 esterase inhibitor, two endogenous regulators of the classical and lectin pathway of complement activation. The combined data underscore the versatility of *B. recurrentis* to effectively evade innate and adaptive immunity, including serum resistance. Thus, the present study elucidates a new mechanism of *B. recurrentis* important for its evasion from complement attack and will be helpful for the development of new drugs against this fatal infection.

## Introduction

*B. recurrentis,* the causative agent of louse-borne relapsing fever is transmitted to humans by contamination of abraded skin with either hemolymph from crushed, infected lice (*Pediculus humanus humanus*) or excreted feces thereof [1], [2]. The last century has seen multiple epidemics of louse-borne relapsing fever in Europe, with high mortality rates of up to 40%. Louse-borne relapsing fever has been epidemic in Africa throughout the 20^th^ century with foci persisting in the highlands of Ethiopia [3], [4]. Clinically, louse-borne relapsing fever is characterized by a 5- to 7-day incubation period followed by one to five relapses of fever, and spirochetemia [5], [6]. Spontaneous mortality remains as high as 2–4% despite antibiotics, with patients suffering from distinctive hemorrhagic syndrome and/or Jarish-Herxheimer reactions [7].

To survive in human tissues, including blood, *B. recurrentis* has to escape innate and adaptive immune responses. Complement is a major component of first line host defense with the potential to eliminate microbes. However, pathogens have evolved strategies to evade complement-mediated lysis, either indirectly, by binding host-derived regulators to their surface or directly, by expressing endogenous complement inhibitors [8], [9]. In fact, we and others have recently demonstrated that tick- and louse-borne pathogens, i.e. *B. hermsii* and *B. recurrentis,* specifically bind complement regulatory proteins, i.e. CFH and CFHR-1, via their outer surface lipoproteins FhbA, BhCRASP-1 and HcpA, respectively [10]–[14]. Surface bound CFH was shown to interfere with the alternative complement pathway by inhibiting complement activation via accelerating the decay of the C3 convertase and inactivating newly formed C3b [15], [16].

However, complement may also attack pathogenic bacteria via the classical pathway, i.e. by interacting with previously bound antibodies, resulting in deposition of the membrane attack complex on the surface of bacteria and their final death [17]. The classical pathway is initiated by the binding and activation of the C1 complex, consisting of C1q, C1r and C1s. C1q can bind to clustered IgG and IgM bound to the surface of bacteria, and also directly to many bacteria through lipoteichoic acids or other structures [18], [19]. When C1q binds, its associated proteases, C1r and C1s, become activated and form the activated C1 complex, which cleaves C4 and C2 to generate the C3 convertase. The lectin pathway is initiated when mannose-binding lectin (MBL) or ficolins bind carbohydrates on the surface of a microbe [20]. A key endogenous regulator of the classical and lectin pathway is serum-derived C4b-binding protein (C4bp). C4bp is a cofactor in factor I-mediated cleavage of C4b to C4d and interferes with the assembly and decay of the C3-convertase (C4bC2a) of the classical and lectin pathway [21], [22]. It was recently shown that acquisition of the regulators CFH and C4bp on the surface of *B. recurrentis* and *B. duttonii* contributes to serum resistance *in vitro* [17]. However, the respective receptors on the spirochetal surface have not been identified.

It was thus the aim of the present study to identify and characterize the putative receptor for C4bp of *B. recurrentis* and *B. duttonii.* Here, we show for the first time that *B. recurrentis* and *B. duttonii* express a novel potential outer surface lipoprotein, which specifically binds C4bp and in addition C1-Inh. The finding that pathogen-bound C4bp retains its co-factor activity suggests that this process contributes to the exceptional resistance of the two spirochetes species to bactericidal activity of human serum.

## Materials and Methods

### Bacterial strains and growth conditions

Relapsing fever spirochetes *B. recurrentis* strains A1 and A17, *B. hermsii* (ATCC35209) strain HS1, *B. duttonii* strain LA, *B. parkeri* RML, *B. turicatae* RML (provided by Tom Schwan, Rocky Mountain Laboratories) and the Lyme disease spirochete *B. burgdorferi* strains ZS7 and B313, a clonal mutant of B31 lacking all linear and circular plasmids with the exception of cp32-1, cp32-2, cp32-4, cp26 and lp17 [23], [24], were cultivated in BSK-H complete medium (Bio&Sell, Feucht, Germany) supplemented with 5% rabbit serum (PAN Biotech, Freiburg, Germany) at 30°C. Bacteria were harvested by centrifugation and washed with phosphate-buffered saline. The density of spirochetes was determined using dark-field microscopy and a Kova counting chamber (Hycor Biomedical, Garden Grove, CA). *E. coli* JM109 were grown at 37°C in LB medium.

### Human plasma and sera

All human plasma and serum samples used in this study were purchased from the Heidelberg University blood bank. Human plasma obtained from 20 healthy, anonymous blood donors without known history of spirochetal infections were pooled and used as source for C4bp. Nonimmune human serum (NHS) was acquired from healthy donors with no prior history of *Borrelia* spp. infection. Factor B-depleted human serum was purchased from Complement Technology, Inc. (http://www.ComplementTech.com).

### Complement proteins

C4bp protein was purified from pooled human plasma by barium citrate precipitation as described [25]. Briefly, following extensive dialysis the solution was subjected to ion exchange chromatography using Q-Sepharose (GE Healthcare) and proteins were eluted with a gradient of 0 – 2 M NaCl. C4b, C1-Inh and factor I were purchased from Calbiochem. Purified C4bp, C1-Inh and BSA were conjugated to biotin with No-Weigh Biotin-NHS (Pierce Biotechnology).

### Isolation and cloning of the receptor for C4bp, construction of expression plasmids and production of recombinant proteins

Isolation of the C4bp binding protein of *B. recurrentis* was carried out by co-immunoprecipitation. Whole cell lysates of *B. recurrentis* were prepared as described elsewhere with minor modifications [10]. Briefly, cultures were grown at 33°C in modified BSK medium to the late-log phase and harvested by centrifugation at 6.000×g for 10 min at 4°C. The resulting pellets were washed twice with PBS, resuspended in ice-cold 50 mM Tris-HCl (pH 7.5), 25 mM KCl, 5 mM Mg_2_Cl, 1mM EGTA, 0.5% NP40 and rotated for 1 h at 4°C. Cell debris were removed by centrifugation and for pre-clearing lysates were incubated with protein G sepharose (GE Healthcare) for 1 h at 4°C. For immunoprecipitation pre-cleared *B. recurrentis* lysates were incubated with protein G Sepharose previously loaded with anti-C4bp antibody and purified human C4bp for 12 h at 4°C with gentle agitation. After washing in 50 mM NaH_2_PO_4_, 300 mM NaCl, 10 mM imidazole (pH 8) bound proteins were eluted with 2x SDS sample buffer (Serva) and subjected to 14% Tris/Tricine SDS-PAGE under reducing conditions. Immunoprecipitates were separated by SDS-PAGE and visualized by staining with colloidal Coomassie (Pierce/Thermofisher, Bonn, Germany). The selected protein band of 40 kDa was cored from the gel and subjected to MALDI mass spectrometric analysis as previously described [26]. Recently, the genome of the selected *B. recurrentis* strain A1 was sequenced [27]. The identified peptide matched an open reading frame of 1071 bp of the *B. recurrentis* A1 genome, named *cihC*. The gene encoding CihC was amplified by PCR using primers CihC F and CihC R (Table 1), cloned into pGEM-T Easy vector (Promega, Mannheim, Germany) and sequenced by using the BigDye terminator cycle sequencing kit (PE Applied Biosystems). The resulting plasmid pGEM-BrCihC was used as template for construction of expression plasmids by PCR amplification. For recombinant full-length CihC protein, primers CihC Bam and CihC HincII were used. For N- and C-terminal deletion mutants, these primers were applied in combination with CihCΔ83F, CihCΔ122F, CihCΔ160F, CihCΔ149R, CihCΔ190R, CihCΔ260R, and CihCΔ294R (Table 1) resulting in recombinant proteins CihC_Δ20–260_, CihC_Δ83–294_, CihC_Δ122–294_, CihC_Δ20–190,_ CihC_Δ83–149,_ and CihC_Δ160–294_, respectively. The ORF encoding CihC of *B. duttonii* (CihC_BD_) was amplified using genomic DNA of *B. duttonii* strain La in combination with oligonucleotides CihC Bam and CihC Hinc. After digestion with restriction enzymes BamHI and HincII, PCR fragments were ligated in frame into the His_6_-tag encoding sequence into vector pQE-30Xa (Qiagen, Hilden, Germany). For expression of N-terminal His-tagged fusion proteins, the plasmids were transformed into *E.coli* strain JM109 and recombinant proteins were purified as recommended by the manufacturer (Qiagen).

**Table 1:** Oligonucleotides used in this study.

Primer	Sequence (5′ to 3′)	Purpose of Use
CihC F	GGA GGA AAA TGG ATC GAT GAA GAG ACA ATG	amplification of *cihC*
CihC R	ATT TAA GCT ATC TGC CAT TC	amplification of *cihC*
CihC Bam	TAT TGG ATC CGA TTT ATT ATT TGA CGA AG	generation of expression plasmid
CihC Hinc	ATT TAA GCT ATC TGC CAT TC	generation of expression plasmid
CihCΔ83F	GGA CAA CAG GGA TCC ATA G	construction of deletion mutant
CihCΔ122F	GAG ATT AGT AAG GGA TCC AAA GAG	construction of deletion mutant
CihCΔ160F	GAA GGA TCA GGA TCC GGT GGA	construction of deletion mutant
CihCΔ149R	GTG TCG ACA GTT ATG TTG TAC CG	construction of deletion mutant
CihCΔ190R	ATA TTC ATA GTC GAC TCA ATC TTC	construction of deletion mutant
CihCΔ260R	CAA AAG TCG ACT AAA GTT CTT GTG CTA GC	construction of deletion mutant
CihCΔ294R	CTT GGT CGA CTA AAT AGC CCT GTA AAG	construction of deletion mutant
CihC Prom	AAA AGG ATC CAC AAT TAC TTA TAC	construction of pCihC
CihC SphI	GTA AAT TTG CAT GCT TGC TTA AGA G	construction of pCihC

### Production of monoclonal antibodies

Monoclonal antibody BR2, directed against CihC was generated by immunization of Balb/c mice with whole cells of *B. recurrentis* A1 according to a method described elsewhere [28]. All animal research was approved in advance by the Laboratory Animal Committee of the University of Heidelberg (RP Karlsruhe 35-9185.82/A-25/07). The animals were kept in a filter cabinet and given food and water *ad libitum*, with all maintenance performed according to German animal welfare guidelines.

### SDS-PAGE, ligand affinity blot and Western blot analysis

To prepare whole cell lysates *Borrelia* were centrifuged and washed three times with PBS. Cells were resuspended in BugBuster Master Mix (Merck) and lysed for 5–10 min on ice. Borrelial whole cell lysates (15 µg) or purified recombinant CihC proteins (200 ng) were subjected to Tris/Glycine-SDS-PAGE under reducing conditions and transferred to nitrocellulose as previously described [29]. Briefly, after transfer of proteins onto nitrocellulose, nonspecific binding sites were blocked using 5% (w/v) dried milk in TBS (50 mM Tris-HCl pH 7.4, 200 mM NaCl) for 2 h at room temperature (RT). Subsequently, membranes were rinsed two times in TBS and incubated for 1 h at RT with NHS (1:1 diluted in TBS) or purified C4bp. Membranes were washed four times with 50 mM Tris-HCl pH 7.5, 150 mM NaCl, 0.2% Tween20 (TBST) and incubated for 1 h with either peroxidase-conjugated anti-C1-Inh (Linaris) or anti-C4bp antibody (Quidel, San Diego). Following four washes with TBST, blot strips were incubated with a secondary peroxidase-conjugated anti-mouse IgG antibody (Dako, Glostrup, Denmark) for 1 h at RT. Detection of bound antibodies was performed using the enhanced chemiluminescence ECL Western blotting detection reagent and ECL Hyperfilms (GE Healthcare, Amersham).

For Western blot analysis, membranes were incubated for 1 h at RT with either anti-C4c antiserum (Dako), anti-C1s (Atlantic antibody), anti-CihC (mAb BR2) or anti-flagellin (mAb LA21) monoclonal antibodies [30]. For detection of purified recombinant CihC full-length protein and deletion mutants, the anti-His_6_-tag (Calbiochem) antibody was employed.

### Southern blotting

Southern blotting of total genomic DNA was done as previously described [31]. Briefly, 250 ml of *Borrelia* cultures were centrifuged, washed twice in PBS and resuspended in 9 ml of TE (10 mM Tris pH 7.5, 1 mM EDTA) buffer. Subsequently, 20% SDS (1 ml) and 20 mg/ml proteinase K (50 µl) was added and incubated for 1 h at 37°C. NaCl (5 M) and Hexadecyl-trimethyl-ammonium-bromide (10%) was added followed by incubation for 10 min at 65°C. DNA was extracted twice with phenol-chloroform-isoamyl ethanol (25∶24∶1) and DNA was precipitated with 0.6 volume of isopropanol. The precipitates were washed with 70% ethanol and resuspended in H_2_O. 10 µg of total genomic borrelial DNA was prepared as agarose blocks, loaded into the agarose gels and fractionated by pulse-field gel electrophoresis (PFGE) in combination with the CHEF-DR II System (Bio-Rad, Germany). Hybridization with a random primed *cihC* gene probe was conducted as described [32].

### Immunofluorescence analysis

Spirochetes (1×10^7^) were washed with Tris buffer (30 mM Tris, 60 mM NaCl, pH 7.4) and incubated with mAb directed against CihC (mAb BR2) or flagellin (mAb LA21) for 1 h at RT. Spirochetes were then washed with Tris buffer/0.1% BSA, spotted on coverslips and allowed to air-dry for 1 h. After methanol fixation, samples were dried for 15 min and incubated for 1 h in a humidified chamber with Cy3-labeled rabbit anti-mouse IgG (1/200, Dianova). Cells were visualized at a magnification of 1000x using a Nikon Eclipse 90i upright automated microscope and images were obtained using a Nikon DS-1 QM sensitive black and white CCD camera at a resolution of 0.133 µm/pixel.

### In situ protease treatment of spirochetes

Cells of *B. recurrentis* strain A1 were treated with proteases using a modified, previously described method [33]. Briefly, intact borrelial cells were incubated with either proteinase K or trypsin to a final concentration of 0 -12.5 µg/ml. Borrelial cells were then lysed and equal volumes (20 µl) were separated by SDS-PAGE (13%). Proteins were visualized by Western blotting using specific monoclonal antibodies.

### C4bp cofactor assay

Functional activity of C4bp was analyzed by measuring factor I-mediated conversion of C4b to iC4b. Either 100 µl of CihC (0.5 µg/well) or intact *B. recurrentis* A1 spirochetes were coated onto microtiter plates (MaxiSorp, Nunc) and incubated with purified human C4bp (50 µg/ml) for 1h at RT and after washing, C4b (4 µg/ml) and factor I (2 µg/ml) were added and incubated at 37°C for up to 2 h. Supernatants were removed from the wells, subjected to SDS-PAGE (10%) under reducing conditions and transferred to a nitrocellulose membrane. Degradation of C4b was evaluated by using a rabbit anti-C4c antibody (DAKO) followed by a peroxidase-conjugated goat anti-rabbit IgG.

### Complex-formation assay of *B. recurrentis*–bound C1-Inh

The protease inhibitory activity of C1-Inh bound to the borrelial surface was examined by detection of SDS-insoluble complexes of C1-Inh and C1s protease. To opsonize cells with C1, 10^8^ *B. recurrentis* cells were incubated with 10% NHS for 1 h at 30°C. After washing, cells were treated with 1 µg biotinylated C1-Inh for 1 h at 30°C. Following three washes, cells were lysed and the borrelial whole cell preparations were subjected to SDS-PAGE (7.5%) under non-reducing conditions. Proteins were transferred to nitrocellulose membranes and probed with either peroxidase-conjugated streptavidin or goat anti-C1s (Atlantic antibody) followed by a peroxidase-conjugated rabbit anti-goat IgG.

### Construction of a shuttle vector for transformation of *B. burgdorferi* B313

The CihC encoding *cihC* gene including its native promoter region was amplified by PCR using primers CihC Prom and CihC SphI. The resulting amplicon was cloned into pBSV2 yielding shuttle vector pCihC. Transformation of *B. burgdorferi* B313 and characterization of transformants was previously described [11]. Expression of CihC of transformed *B. burgdorferi* B313 was determined by Western blot, whole cell ELISA and immunofluorescence analysis, using mAb BR2. High-passage, non-infectious *B. burgdorferi* strain B313 were grown in 100 ml BSK medium and harvested at mid exponential phase (10^8^ cells/ml). Electrocompetent cells were prepared as described previously [26] with slight modifications. Briefly, 50 µl aliquots of competent *B. burgdorferi* strain B313 cells were electroporated at 12.5 kV/cm in 2-mm cuvettes with 10 µg of plasmid DNA. For control purpose *B. burgdorferi* strain B313 cells also were transformed with pBSV2 vector alone. Cells were immediately diluted into 10 ml BSK medium and incubated without antibiotic selection at 30°C for 48 to 72 h. Bacteria were then diluted into 100 ml BSK medium containing kanamycin (30 µg/ml) and 200 µl aliquots were plated into 96-well cell culture plates (Corning) for selection of transformants. After three weeks, wells were evaluated for positive growth by color change of the medium, confirmed by dark-field microscopy for the presence of motile spirochetes. The *cihC* gene of transformed *B. burgdorferi* B313 was detected by PCR using oligonucleotides CihC F and CihC SphI. Ectopic CihC expression was analyzed using immunofluorescence microscopy and ELISA in combination with mAb BR2. In addition, ectopically expressed CihC was analyzed by ligand affinity blotting and flow cytometry with regard to its capacity to acquire C4bp and C1-Inh.

### Flow cytometry

Briefly, 10^7^ *B. recurrentis* A1, *B. duttonii* La, *B. burgdorferi* B313/vc and B313/pCihC cells were washed twice with PBS, blocked for 15 min at RT with PBS/10% BSA, and incubated with 10 µg/ml of biotinylated C4bp or C1-Inh in FACS-buffer (PBS/1% BSA) for 1 h at RT. As a negative control, spirochetes were incubated with the same concentration of biotinylated BSA. Cells were washed three times, stained with phycoerythrin (PE) labeled streptavidin (Bio-Rad) and were then fixed with 1% paraformaldehyde overnight and analyzed using a FACS-Calibur and the CellQuest software (BD Biosciences).

### Serum susceptibility testing of *Borrelia* strains

The serum susceptibility of mock-transformed *B. burgdorferi* B313 (*B. burgdorferi* B313/vc) and transformed *B. burgdorferi* B313 (*B. burgdorferi* B313/pCihC) was assessed using a survival assay. Cells grown to mid-logarithmic phase were harvested, washed and approximately 3×10^7^ spirochetes were resuspended in BSK-H medium supplemented with either 50% factor B-depleted human serum (NHS_-B_) or 50% heat inactivated factor B-depleted human serum (hiNHS_-B_). Cells were incubated in Eppendorf tubes at 30°C for 2 days. At day 0, 1, and 2, cells were washed in 0.85% NaCl, transferred to microtiter plates and incubated with SYTO9 (Molecular Probes, Invitrogen) as recommended by the manufacturer. Subsequently, relative growth of spirochetes as compared to day 0 was determined by measuring the fluorescence intensity at 530 nm (excitation 485 nm) on a microtiter plate reader (Victor2 plate reader, Perkin Elmer).

### ELISA

For whole cell ELISA, approximately 10^8^ spirochetes (*B. burgdorferi* B313/vc and B313/pCihC) were washed twice, resuspended in PBS and immobilized on microtiter plates overnight at 4°C. The wells were washed with PBS/0.05%Tween, blocked with PBS/5% BSA and were then incubated with the CihC-specific mAb BR2 or the flagellin-specific mAb LA21 followed by a peroxidase-conjugated sheep anti mouse IgG. Substrate reaction was performed with o-phenyldiamine dihydrochloride (Sigma-Aldrich) and absorbance was measured at 492 nm.

### Statistical analysis

Statistics were analyzed with the unpaired Student's t-test, P values less than 0.05 were considered significant.

### Nucleotide sequence deposition

The *cihC* gene sequences of *B. recurrentis* and *B. duttonii* reported in this paper have been deposited in the EMBL/GenBank data bases under the following accession numbers: FN552439 and FN552440, respectively.

## Results

### Cloning and characterization of the receptor for C4bp and C1-Inh

**Figure 1:**
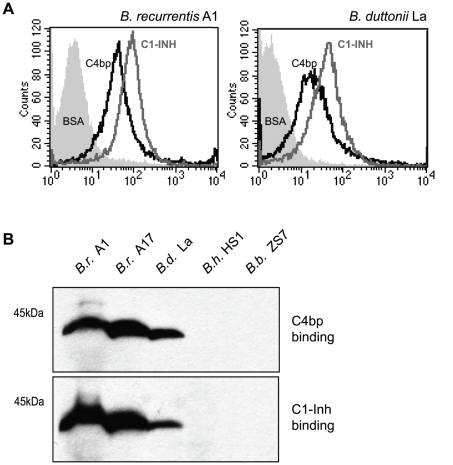
Binding of C4bp and C1-Inh to the spirochetal surface. A) Intact *B. recurrentis* A1 (left panel) and *B. duttonii* La (right panel) spirochetes were incubated with biotinylated human C4bp, C1-Inh or as a negative control with biotinylated BSA, followed by PE-labeled streptavidin and were then analyzed by flow cytometry. B) Binding of human C4bp and C1-Inh to a≈40 kDa protein. Whole cell lysates of *B. recurrentis* strains A1 (*B.r.* A1) and A17 (*B.r.* A17), and *B. duttonii* La (*B.d.* La) were separated by SDS-PAGE, transferred to nitrocellulose membrane and incubated with purified human C4bp (upper panel) or NHS (lower panel). Membranes were probed with anti-C4bp antiserum followed by peroxidase-conjugated secondary antibody (upper panel) or with C1-Inh-specific peroxidase-conjugated IgG (lower panel). As a control, cell lysates of *B. hermsii* HS1 (*B.h.* HS1) and *B. burgdorferi* ZS7 (*B.b.* ZS7) were included.

**Figure 2:**
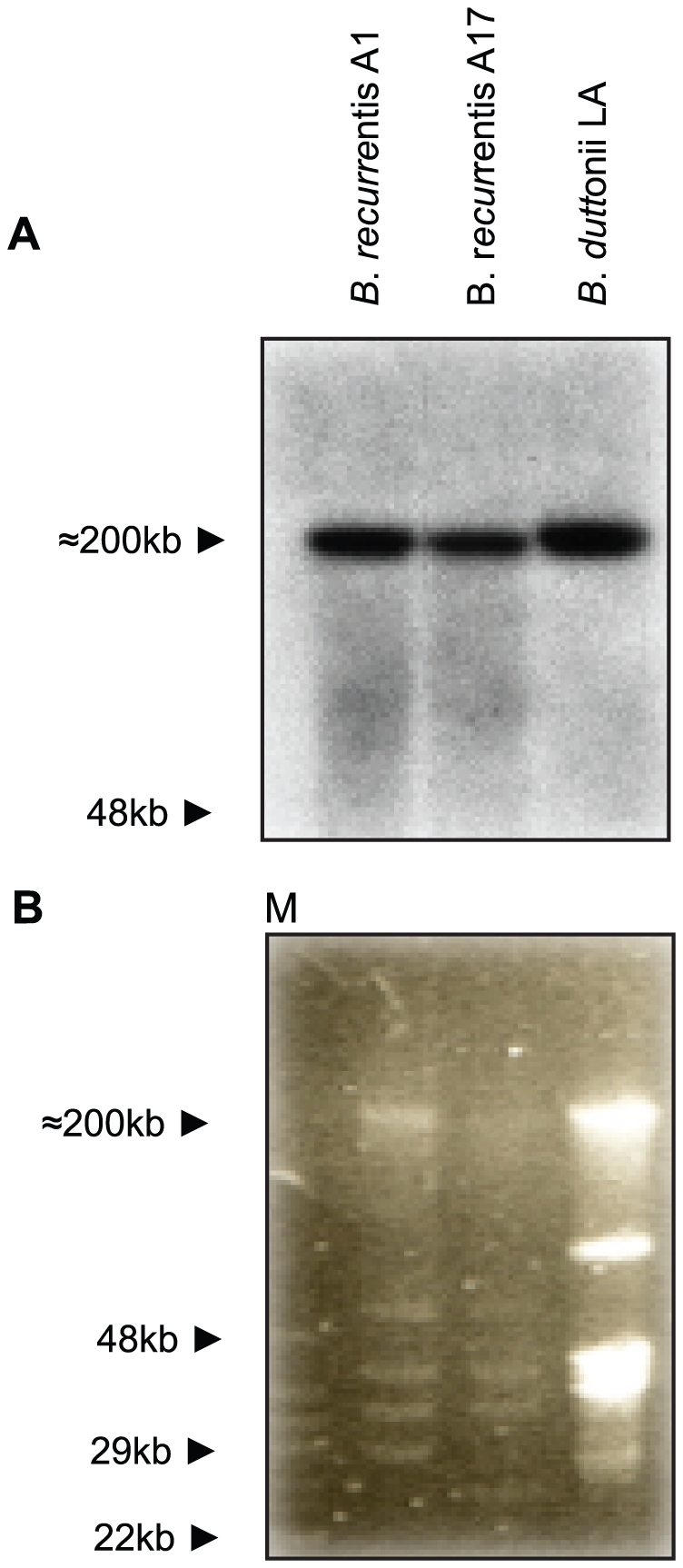
Genomic localization of *cihC*. A) Genomic DNA of indicated relapsing fever *Borreliae* was separated by PFGE and probed with a full-length ^32^P labelled *cihC* gene probe. Linear plasmids are indicated by arrows. B) PFGE patterns of the indicated *Borrelia* strains.

**Figure 3:**
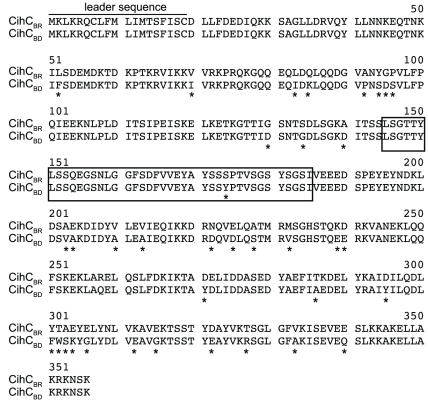
Alignment of the deduced amino acid sequences of CihC_BR_ (*B. recurrentis* A1) and CihC_BD_/BDU_1 (*B. duttonii* La). Divergent residues are marked with asterisks. The putative ligand binding site for human C4bp and C1-Inh is represented by the open box.

To verify acquisition of C4bp onto the outer surface *B. recurrentis* and *B. duttonii* spirochetes were incubated with biotinylated human C4bp and analyzed by flow cytometry. Both strains were found to acquire C4bp onto their surfaces (Fig. 1A). By applying ligand affinity blot analysis for detection of C4bp-binding molecules, a protein of about 40 kDa was identified in *B. recurrentis* and *B. duttonii*, but not in *B. hermsii* and *B. burgdorferi* (Fig. 1B). In addition, *B. recurrentis* and *B. duttonii* are capable of binding the complement regulator C1-Inh (Fig. 1). To isolate and characterize the receptor for C4bp, cell lysates of *B. recurrentis* A1 were incubated with C4bp and added to Protein G Sepharose coupled anti-C4bp immune serum. The co-precipitating protein of approximately 40 kDa was analyzed by mass spectrometry and the peptides generated matched an open reading frame of 1071 bp on the genome of *B. recurrentis* A1 [27]. The open reading frame encoded for a putative lipoprotein with a calculated molecular mass of 40.4 kDa. The encoding gene was designated *cihC* (complement inhibition via C4bp). Pulse-field gel electrophoresis and hybridization analysis revealed that the *cihC* gene represents a single genetic locus in *B. recurrentis* and *B. duttonii* that maps to a larger plasmid of approximately 200 kb (Fig. 2) [27], [34]. Neither the tick-borne relapsing fever strains of *B. parkeri*, *B. hermsii* and *B. turicatae* nor *B. burgdorferi*, the causal agent of Lyme disease, hybridized with the *cihC* probe (data not shown). Isolation of the homologous *B. duttonii* gene revealed 91% amino acid sequence similarity with that of *B. recurrentis* (Fig. 3). Lescot et al. previously identified the *cihC* gene of *B. duttonii* as a p35-like antigen (BDU_1) exhibiting similarity to the *B. burgdorferi* fibronectin-binding lipoprotein BBK32. In contrast to our observation the homologous gene in *B. recurrentis* was not detected [27]. Interestingly, our preliminary studies indicated that recombinant CihC of *B. duttonii* and *B. recurrentis* binds fibronectin (unpublished). A BLAST search failed to detect any other protein with significant homology, indicating that the two genes/proteins are restricted to these highly related species of *Borrelia*.

### Surface exposure and protease sensitivity of the receptor for C4bp and C1-Inh

**Figure 4:**
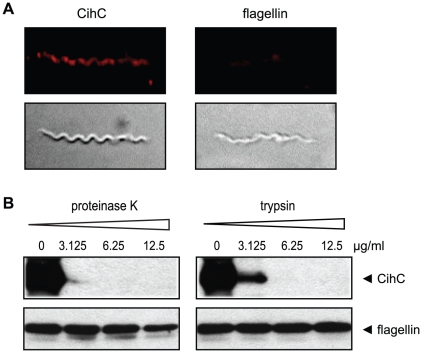
Surface localization of CihC. A) Immunofluorescence analysis of *B. recurrentis* A1 after incubation with mAb specific for CihC (BR2) followed by rabbit anti mouse Cy3-conjugated IgG. Antibodies directed against flagellin were included as a non-surface accessible control protein. The corresponding differential interference contrast image is depicted in the lower panel. B) Proteinase K and trypsin treatment affects surface expression of native CihC. *B. recurrentis* cells were incubated with the indicated concentrations of proteinase K and trypsin, lysed, immunoblotted, and probed with either anti-CihC mAb BR2 (upper panel) or with anti-flagellin mAb LA21 (lower panel).

To determine whether CihC is surface exposed, immunofluorescence assays were performed using mAb BR2 specific for CihC. *B. recurrentis* spirochetes were incubated sequentially with mAb BR2 and rabbit anti-mouse Cy3-conjugated antibody (Fig. 4A). Epifluorescence microscopy revealed that *B. recurrentis* organisms expressed CihC on their outer surface in a patch-like manner. Controls incubated with the secondary antibody alone were negative (not shown).

To further confirm surface localization of CihC, *B. recurrentis* organisms were treated with either proteinase K or trypsin and subjected to Western blot analysis. As shown in Figure 4B, a significant reduction was observed for CihC after 2 h of incubation with proteinase K at concentrations ≥3 µg/ml. Upon treatment of the spirochetes with trypsin, a more restricted protease, only higher amounts (≥6 µg/ml) yielded complete degradation of CihC. The mouse mAb LA21 directed against the periplasmic FlaB protein was used in this experiment as a internal control to confirm that the fragile spirochetal outer membrane was not damaged (Fig. 4B, lower panels). These data indicate that CihC is exposed at the outer surface of *B. recurrentis*.

### Localization of the binding domains of the C4bp and C1-Inh receptor

**Figure 5:**
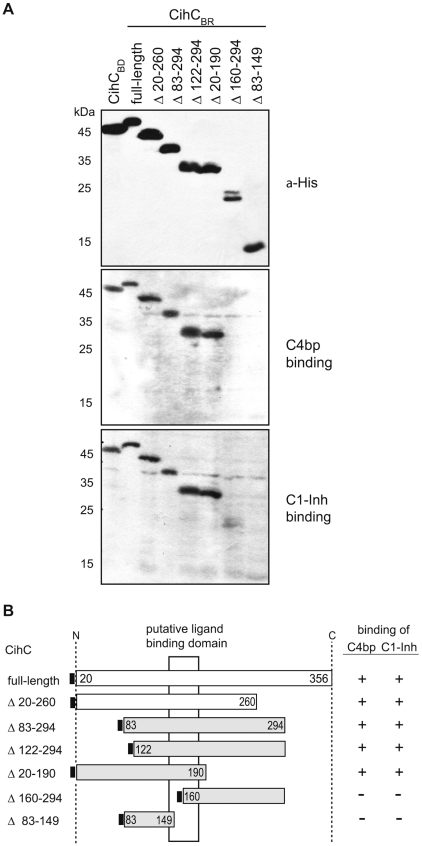
Mapping of the CihC domain interacting with C4bp and C1-Inh. A) Whole cell lysates of *E. coli*, expressing the recombinant, His-tagged CihC_BR_, CihC_BD_ and the indicated CihC_BR_ deletion mutants were separated by SDS-PAGE, transferred to nitrocellulose and were either probed with an anti His-tag mAb (upper panel) or subjected to ligand affinity blotting using NHS as a source for human C4bp and C1-Inh. B) Diagrammatic representation of native and expressed recombinant CihC_BR_ and CihC_BD_ proteins and their binding characteristics for C4bp and C1-Inh as determined by ligand affinity blot analysis. Numbers refer to amino acid residues.

To localize the putative domain(s) of CihC that bind to C4bp and C1-Inh, a number of CihC deletion mutants with distinct N- or C-terminal truncations were constructed (Fig. 5A). Protein expression was confirmed by using a His-tag specific antibody and all recombinant proteins exhibited the predicted size. Screening for C4bp and C1-Inh binding by ligand affinity blotting revealed that from the polypeptide preparations tested, full-length CihC (residues 20 to 356) and all truncated versions employing the central protein domain (amino acid residues 145 – 185) similarly retained C4bp and C1-Inh binding activity (Fig. 5B). These results suggest that CihC contained a central region that bound to both human complement regulators.

### C4bp retains cofactor activity when bound to its receptor

**Figure 6:**
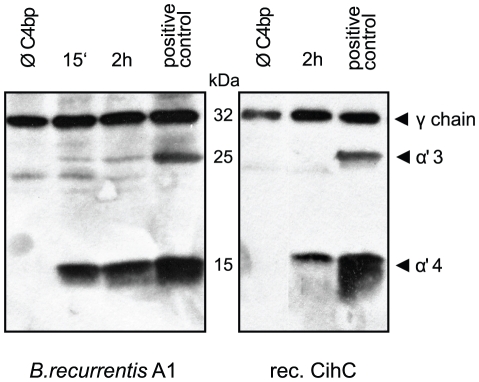
Cofactor activity of C4bp bound to CihC and *B. recurrentis*. A) Functional activity of C4bp was analyzed by measuring factor I-mediated conversion of C4b to iC4b. C4bp bound to the surface of intact *B. recurrentis* spirochetes (left panels) or to CihC coated microtiter plates (right panels) was incubated with C4b and factor I. Reaction mixtures were separated by SDS-PAGE and transferred to nitrocellulose membrane. C4b alpha chain degradation products were detected using polyclonal anti C4c antibody followed by peroxidase-conjugated secondary antibody. As a positive control C4bp was directly added to C4b and factor I.

Inactivation of complement component C4b occurs by factor I mediated cleavage of the C4b alpha chain. To assess whether C4bp maintains this cofactor activity when attached to the surface of intact *B. recurrentis* spirochetes were coated with purified human C4bp and incubated with C4b and factor I. The supernatant was subjected to SDS-PAGE and C4b alpha chain degradation products were detected by immunoblot analysis. As shown in Figure 6 (left panel), binding of C4bp to the cell surface resulted in α4 and α3 degradation products of 15 kDa and 25 kDa, respectively. In contrast, *B. recurrentis* spirochetes alone did not promote factor I-mediated cleavage of C4b demonstrating that louse-borne relapsing fever spirochetes lack endogenous C4b cleaving activity. Similarly, C4bp bound to immobilized recombinant CihC protein efficiently mediated C4b processing via factor I, as indicated by the appearance of a α4 fragment (Fig. 6, right panel). *B. recurrentis* or CihC preincubated with C4bp and C4b in the absence of factor I did not promote cleavage of C4b (data not shown). These findings demonstrate that CihC-associated C4bp retains its cofactor activity and may lead to accelerated disintegration of the C3 convertase (C4bC2a) of the classical complement activation pathway.

### C1-Inh bound to *B. recurrentis* exhibits complement inhibitory activity

**Figure 7:**
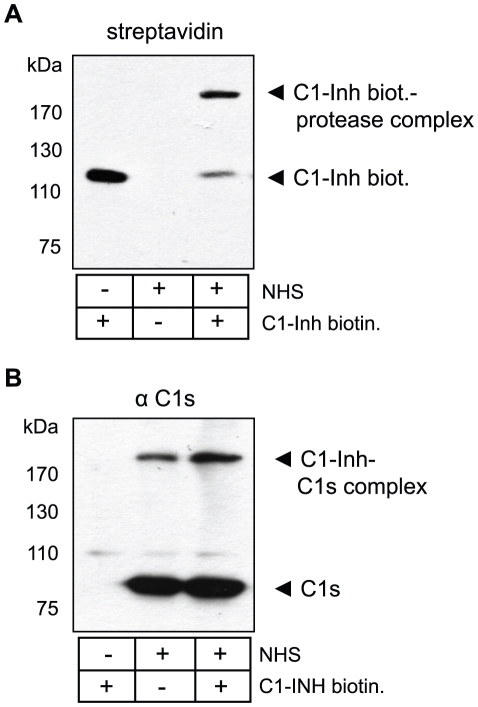
Complex formation of C1-Inh bound to *B. recurrentis*. Protease inhibitory activity was evaluated by the formation of C1-Inh/protease SDS-insoluble complexes. Spirochetes were incubated with NHS, washed and subsequently treated with biotinylated human C1-Inh. Cells were lysed, immunoblotted and probed with either A) peroxidase-conjugated streptavidin or B) anti-C1s antibodies followed by peroxidase-conjugated IgG.

The protease inhibitor C1-Inh reacts with its complement target proteases such as C1s and C1r to form high molecular weight SDS resistant complexes [35]. We examined the formation of these covalent C1-Inh-protease complexes as an index for the protease inhibitory activity of CihC-associated C1-Inh. Intact *B. recurrentis* cells were preincubated in NHS as source for C1 and biotinylated C1-Inh was applied. Subsequently, cells were washed extensively to remove unbound C1-Inh, lysed and subjected to immunoblotting. As shown in Figure 7A, biotinylated C1-Inh acquired by *B. recurrentis* formed complexes on the spirochetal surface as indicated by the occurrence of a high molecular weight band at >170 kDa. To identify the constituent protease of these complexes, immunoblot analysis using C1s-specific antiserum was employed revealing C1s is a component of the >170 kDa large complex (Fig. 7B). Exogenously applied biotinylated C1-Inh as well as serum-derived C1-Inh formed the respective complexes with C1s. These data suggest that C1-Inh bound to the surface of *B. recurrentis* retains its functional activity and thus, by inactivating C1s protease, exhibits complement inhibitory activity.

### Expression of the receptor for C4bp and C1-Inh increases resistance to complement-mediated killing

**Figure 8:**
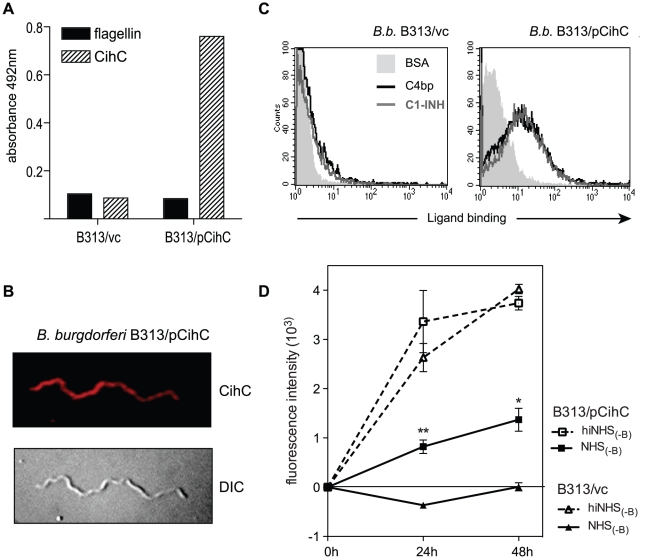
Ectopic expression of CihC in serum-sensitive *B. burgdorferi* B313. A) Expression and surface localization of CihC by transformed *B. burgdorferi* B313 was analyzed by whole cell ELISA using CihC-specific mAb BR2 and as control, the flagellin-specific mAb LA21. B) Binding of C4bp and C1-Inh to B313/vc and transformed B313/CihC cells was analyzed by FACS analysis. C) Immunfluorescence analysis using CihC-specific mAb BR2 followed by rabbit anti-mouse Cy3-conjugated IgG. The corresponding differential interference contrast image is shown in the lower panel. D) For human serum susceptibility assay, mock-transformed *B. burgdorferi* B313 (B313/vc) and *B. burgdorferi* B313 transformed with the *cihC* gene (B313/pCihC) were incubated in the presence of 50% factor-B depleted human serum (NHS_-B_) or heat-inactivated factor B-depleted human (hiNHS_-B_) serum at 30°C for 48 h. Cells were stained with a nucleic acid dye and the growth as compared to day 0 was determined by measuring of the fluorescence intensity at 530 nm. Values represent the mean ± SEM of a single experiment performed in triplicate that is representative of three independent experiments. **, P = 0.001; *, P<0.01 for B313/pCihC NHS_(-B)_ at 24h and 48h, respectively, compared to B313/vc NHS_(-B)_.

To test whether CihC of *B. recurrentis* plays an important role in mediating complement resistance, the serum-sensitive *B. burgdorferi* B313 mutant strain was transformed with the shuttle vector pCihC containing the complete *cihC* gene (*B. burgdorferi* B313/pCihC); for control, the pBSV2 vector alone (*B. burgdorferi* B313/vc) was employed. Expression and surface localization of CihC was determined by whole cell ELISA (Fig. 8A) and immunofluorescence (Fig. 8B) analyses using the CihC-specific mAb BR2. Moreover, to ascertain whether the ectopically expressed CihC is capable of recruiting C4bp and C1-Inh to the surface of B313/pCihC flow cytometry was performed (Fig. 8C). The B313/pCihC-transformed isolate but not the mock-transformed B313/vc isolate of *B. burgdorferi* strongly expressed CihC and acquired both complement regulators.

To compare the susceptibility of B313/pCihC and B313/vc to complement-mediated killing, both specimens were subjected to a human serum sensitivity assay. In order to avoid killing of *Borrelia* strains via the alternative pathway of complement activation a factor B-depleted human serum was employed. Accordingly, spirochetes were incubated in factor B-depleted human serum (NHS_-B_) or heat-inactivated factor B-depleted serum (hiNHS_-B_) and spirochetal growth was monitored by uptake of a nucleic acid dye. B313/pCihC and the mock-transformed strain multiplied during the 48 h time interval when incubated with heat-inactivated factor B-depleted serum (Fig. 8D). However, when exposed to NHS_-B_ only B313/pCihC spirochetes could replicate indicating that ectopic expression of CihC renders serum-sensitive *B.burgdorferi* B313 more resistant to complement-mediated lysis. These data suggest a decisive role for CihC in serum resistance of *B. recurrentis* and *B duttonii*.

## Discussion

Bacteria have evolved multiple strategies to interfere with complement-mediated clearance of pathogens by blocking distinct steps of the lytic cascade. Recently, we provided evidence that the louse-borne relapsing fever spirochete *B. recurrentis* selectively inhibits activation of the alternative complement pathway by specifically binding the endogenous complement inhibitor CFH via its lipoprotein HcpA [11]. We now demonstrate for the first time that *B. recurrentis* also expresses a surface receptor specific for C4bp and C1-Inh, two major serum-derived inhibitors of the lectin and classical complement pathways, termed CihC. Genetic and molecular analyses revealed that CihC of *B. recurrentis* is a potential lipoprotein and that *B. duttonii* harbors a homologue of CihC [27]. Upon binding to the pathogen's surface or to recombinant CihC, C4bp retained its cofactor activity for factor I-mediated C4b inactivation. Together with the fact that *B. recurrentis* also expresses HcpA, the presented data suggest that the potential of louse-borne relapsing fever spirochetes to interfere with both, classical and alternative pathways, contributes to their high resistance and pathogenicity in humans.

The correlation between serum resistance of bacteria and cell surface binding of functionally active C4bp has been reported before for a number of pathogenic microorganisms, including the spirochetes *B. recurrentis*, *B. duttonii* and *B. burgdorferi* s.s. (strain IA), the causative agent of Lyme disease [17]. Moreover, when incubated with human serum, *Yersinia enterocolitica*, *Bordetella pertussis*, *Neisseria gonorrhoeae*, *Candida albicans*, *Moraxella catarrhalis*, *Escherichia coli* K1, *Streptococcus pyogenes* and *Yersinia pestis* were also shown to acquire C4bp [36]–[43]. However, the respective receptors for C4bp have only been identified for some bacteria, e.g. *Streptococcus pyogenes*, *Yersinia enterocolitica*, and *Moraxella catarrhalis*, but not for *B. recurrentis*, *B. duttonii* and *B. burgdorferi*. The present data provide evidence that the receptor for C4bp of *B. recurrentis*, CihC is a surface exposed putative lipoprotein. Preliminary Southern Blot analysis and BLASTN search on databases revealed a putative homologue of *cihC* only in *B. duttonii* but not in other spirochetal species suggesting that the gene encoding C4bp receptor is unique to these two *Borrelia* species. To determine whether the *cihC* gene is located either on the chromosome or any of the linear plasmids, PFGE and Southern blotting were performed. The *cihC* gene was localized to a 190 kb linear plasmid adjacent to the previously identified factor H binding *hcpA* gene. Similarly, the *B. hermsii* gene encoding the factor H binding protein FhbA maps to the large linear plasmid of 220 kb [13]. However, further studies are required to resolve this issue for other bacterial pathogens.

To localize the peptide domains of CihC relevant for binding of C4bp and C1-Inh, truncated N- and C-terminal deletion mutants were generated and used for functional analyses. C4bp and C1-Inh binding was not abrogated by N-terminal (amino acid residues 20–121) or the C-terminal (amino acid residues 191–356) deletion mutants of CihC indicating that both, C4bp and C1-Inh, bind to the central domain of CihC. In related studies, *Streptococcus pyogenes* was previously shown to bind C4bp through the N-terminal highly variable region of M-proteins Arp and Sir and similar results were also obtained with the FHA receptor for C4bp of *Bordetella pertussis* [38], [39], [41], [44]. However, the reason for the differential binding domains of the various pathogen receptors for C4bp is not known at present.

Preliminary data indicate that binding of C4bp to CihC ectopically expressed by *B. burgdorferi* B313 cells is independent of ionic strength suggesting a hydrophobic interaction between the receptor and its ligand. Similar findings have been reported before for other pathogens. Thus, interaction of the *Y. enterocolitica* Ail receptor with C4bp was also found to be less sensitive to salt [45]. Moreover, C4bp receptors like Por1A of *N. gonorrhoeae* [42], [46], UspA1/2 of *M. catarrhalis* [36], OmpA of *E. coli* [43] and the M-proteins of *S. pyogenes* bind C4bp in a nonionic fashion. However, further analyses including C4bp deletion constructs are required to solve this issue for CihC.

The present study adds another facet on the versatility of relapsing fever spirochetes to persist in human blood and to evade innate and adaptive immunity. The best-known immune evasion strategy of relapsing fever *Borrelia* is antigenic variation, i.e. the ability to respond to newly generated specific antibodies with a switch to an altered variable major outer surface protein (Vmp). Essentially, the pathogen always stays one step ahead of antibodies. However, while antigenic variation is restricted to Vmps, other surface exposed proteins are stable and antigenic, e.g. the surface-exposed lipoprotein FhbA of *B. hermsii* [12], [13], [47], [48]. In this context it could be speculated that upon binding to CihC, C4bp and C1-Inh inhibit the lectin and classical complement pathway, including the formation of the lytic membrane attack complexes.

In addition to the observed anti-complement activity, *B. recurrentis*-exposed C4bp may exhibit another biological activity relevant for spirochetal serum resistance. This is indicated by the fact that C4bp circulates in plasma as a complex with protein S that in turn binds to negatively charged phospholipids on membranes [49]–[51]. Thus, it is possible that C4bp also promotes adhesion and subsequently hematogenous dissemination by simultaneously binding to *B. recurrentis* and endothelial cells. This assumption is supported by the recent observation that the related fibronectin and glycosaminoglycan binding protein, BBK32, of *B. burgdorferi* mediates endothelial interactions *in vivo*, thereby facilitating microvascular interactions [52]. Similarly, CihC of *B. recurrentis* and *B. duttonii* bound fibonectin and thus could also be involved in the dissemination process of relapsing fever spirochetes.

The assumption that CihC of *B. recurrentis* and probably also CihC/BDU_1 of *B. duttonii* are critically involved in their escape from complement-mediated lysis is further supported by the present finding that ectopic expression of CihC in the serum-sensitive *B. burgdorferi* strain B313 led to a significant increase in resistance to complement mediated lysis. Moreover, binding of C1-Inh, the major inhibitor of several pathways of inflammation in humans, to CihC could be observed. However, the actual role of CihC in the pathogenesis of louse-borne relapsing fever will only be elucidated by *in vivo* studies in a relevant mouse model [53]. Complement resistance in *cihC* transformed *B. burgdorferi* strain was detected in the presence of non-immune factor B-depleted human serum indicating that the lectin/classical pathway of complement activation may be triggered by *Borrelia* structures other than specific antibodies. Indeed, we have shown that C1q and the C1 complex can bind to the surface of *B. recurrentis* in the absence of specific antibodies. Moreover, recognition molecules specific for the lectin pathway (i.e. MBLs and ficolins) could also bind to borrelial carbohydrates and activate MASPs [20], [54]–[58]. MASP-2 is the enzyme component that, like C1s in the classical pathway, cleaves the complement components C4 and C2 to form the C3 convertase C4bC2a, common for activation of both the lectin and the classical pathways. However, it remains to be determined whether C4bp and C1-Inh binding significantly increases *B. recurrentis* spirochetes resistance against complement attack in humans.

In summary, this study is the first to show that *B. recurrentis* and most probably *B. duttonii* express a potential lipoprotein receptor, which selectively binds C4bp and C1-Inh, the endogenous regulators of the classical and lectin complement pathway. Together with the fact, that both spirochetal species also carry a specific receptor for the serum-derived complement inhibitor of the alternative pathway, CFH, the present data emphasize the versatility of *B. recurrentis* and *B. duttonii* to evade lectin/classical and alternative pathways of complement activation. Elucidating the pathological processes underlying relapsing fever will be helpful to design novel regimens for therapeutic treatment of spirochete-induced relapsing fever and to develop potential vaccine candidates.
